# Regenerated Oxidized Cellulose as a Sealant and Adhesive in Endoscopic Endonasal Skull Base Reconstruction

**DOI:** 10.1055/s-0044-1788599

**Published:** 2025-04-15

**Authors:** Ashwin Gajendran Vedhapoodi, Aravind Sabesan, Benazir Ferozkhan, Saravana Selvan Velmurugan, Venkatesan Rajarajan, Baskar Arukavur Radhakrishnan, Kanagaraman Prabhuraman, Bhuvaneswari Natarajan

**Affiliations:** 1 Department of Otorhinolaryngology, Head and Neck Surgery, Government Stanley Medical College, Chennai, Tamil Nadu, India; 2 Department of Neurosurgery, Government Stanley Medical College, Chennai, Tamil Nadu, India; 3 Department of Otorhinolaryngology, Madras Medical College, Chennai, Tamil Nadu, India; 4 Department of Community Medicine, Sri Venkateswaraa Medical College Hospital and Research Centre, Puducherry, Tamil Nadu, India

**Keywords:** Surgicel, regenerated oxidized cellulose, CSF leak, tissue sealant, tissue adhesive

## Abstract

**Introduction** An ideal and long-lasting adhesive and sealant is essential during endoscopic endonasal skull-base surgery to hold the reconstruction intact and prevent cerebrospinal fluid (CSF) permeation until complete healing occurs. Fibrin glue is the most common material used. Regenerated oxidized cellulose (ROC) has not been mentioned in the literature as sealant and adhesive, and, hence, we intended to study this role.

**Objective** To evaluate the role of ROC as tissue sealant and adhesive in the reconstruction of skull-base defects in endoscopic endonasal skull-base surgery.

**Methods** We retrospectively analyzed the medical records of patients who underwent endoscopic endonasal skull-base surgery with skull-base defect and intraoperative CSF leak, for which reconstruction was performed using fibrin glue or ROC, or both, as a sealant and adhesive. The type of sealant and adhesive used and postoperative CSF leak rates with different agents used were analyzed.

**Results** A total of 64 patients were investigated. Fibrin glue alone was used initially in 6 patients, of which 4 (66.6%) experienced postoperative CSF leak. Both fibrin glue and ROC were used in 26 patients, among which 2 (7.6%) exhibited postoperative CSF leak. Regenerated oxidized cellulose alone was used in 24 patients, wherein 2 (8.3%) presented with postoperative CSF leak. Fibrin glue alone was once again used later in the learning curve in 8 patients, of which 2 (25%) experienced postoperative CSF leak ( *p* = 0.002).

**Conclusion** Fibrin glue provides intraoperative watertight seal. Regenerated oxidized cellulose has better intraoperative and long-term sealant and adhesive action in endoscopic endonasal skull-base reconstruction.

## Introduction

The use of expanded endonasal approaches (EEAs) has been increasing due to the refinements achieved in the reconstruction of large skull-base defects and closure of high-flow cerebrospinal fluid (CSF) leaks. The aim of reconstruction is to achieve a watertight seal with total separation between the arachnoid and sinonasal compartments. 1 Various materials have been tested for obtaining successful closure, including acellular dermal allograft 2 and free tissue autografts such as fat, fascia, mucosal free grafts, cartilage, and bone. 3 4 The development of the Hadad-Bassagasteguy flap, a vascular pedicled mucoperichondrial and mucoperiosteal flap based on the posterior septal artery, was a major breakthrough in skull-base reconstruction surgery. 5 6 7 8 9 10 11 Various multilayered reconstruction techniques have been described in literature, such as the gasket seal 9 and bilayer button techniques. 10

Endoscopic skull-base surgery involves the extensive removal of mucosal, soft tissue and bone. Multilayer reconstruction using fat, fascia, and pedicled flaps is now a common practice. Although flap adherence occurs within a few days, complete healing and mucosalization is achieved over several weeks to months. In living organisms, cells to more complex structures are held together in a stable manner by adhesive molecules in specific dimensions and positions. Similarly, an ideal and long-lasting adhesive and sealant is essentially required during endoscopic skull-base surgery to hold the multilayer reconstruction intact and prevent permeation of CSF until complete healing is attained. Numerous studies have been published in favor of as well as against the use of dural sealants. All these studies have discussed the use of fibrin glue and its variants.

Hemostatic agents induce the clotting of blood. Tissue sealants are agents which prevent seepage of body fluids, such as blood and CSF. The adhesives are substances which assist in the adherence of tissues or tissue fixation. Fibrin glue is a tissue sealant with hemostatic and adhesive activity for which it has Food and Drug Administration (FDA) approval. Surgicel (Ethicon, Inc., Raritan, NJ, USA), however, is mentioned only as a hemostatic agent. 12

## Objective

To evaluate the role of regenerated oxidized cellulose (ROC) gauze (Surgicel) as a tissue sealant and adhesive, in addition to its hemostatic role, in the reconstruction of skull-base defects following endoscopic endonasal skull-base surgery.

## Methods

Here, we retrospectively analyzed the medical records of patients who underwent endoscopic endonasal skull-base surgery with intraoperative CSF leak, for which reconstruction was performed using fibrin glue or Surgicel (ROC) or both as sealants/adhesives. The demographic data, approach used, size of the defect, type of intraoperative CSF leak, type of repair performed, type of sealant/adhesive used, and postoperative CSF leak rate with different sealants/adhesives used were analyzed. This retrospective analysis was conducted after obtaining approval from the Institutional Ethics Committee (Ethics committee registration number: ECR/131/Inst/TN/2013/RR-22). The requirement for informed patient consent was waived because the study involved a retrospective analysis of medical records. This study conforms to the Declaration of Helsinki standards. We included all patients who underwent skull-base reconstruction with fibrin glue or ROC or both as sealants/adhesives from January 2018 to June 2023.

### Cardinal Surgical Steps of Reconstruction

**Fig. 1:**
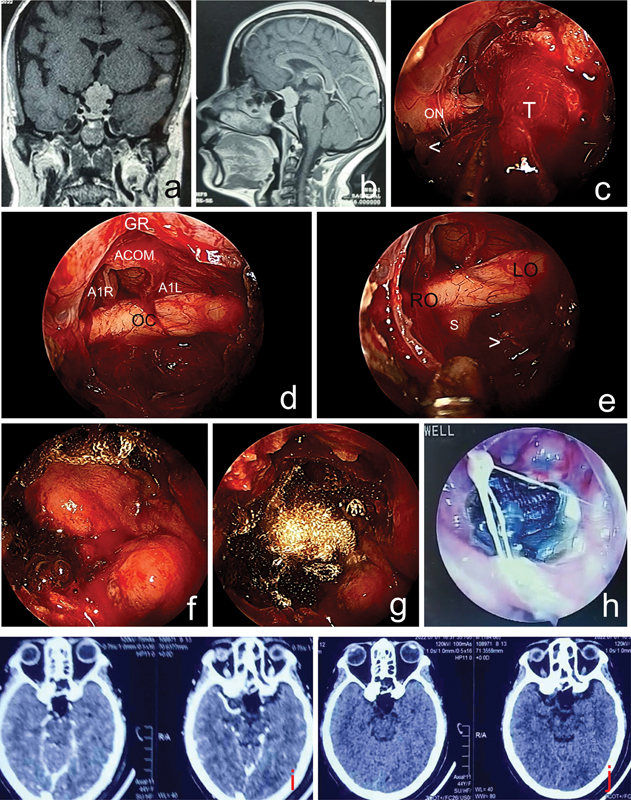
A case of suprasellar meningioma for which endoscopic transplanar, tubercular approach was performed. ( **A,B** ) Preoperative magnetic resonance imaging scans. ( **C** ) Tumor dissection. Abbreviations: T, fibrous tumor; ON, right optic nerve. Note: white (<), right supraclinoid internal carotid artery. ( **D,E** ) Posttumor removal. **Abbreviations:** A1R, A1 segment of right anterior cerebral artery; A1L, A1 segment of left side; ACOM, anterior communicating artery; GR, gyrus rectus; LO, left optic nerve; OC, optic chiasma, RO, right optic nerve, S-P, pituitary stalk. **Note:** white (>), left superior hypophyseal artery. ( **F,G** ) Hadad flap with single layer of Surgicel used mainly at the margins to adhere the flap to the underlying bone. ( **H** ) – One-month postoperative cavity showing the mold of crust and the flap incorporated with a healthy mucosal healing. ( **I,J** ) Postoperative day 1 computed tomography scan showing complete excision of tumor.

**Fig. 2:**
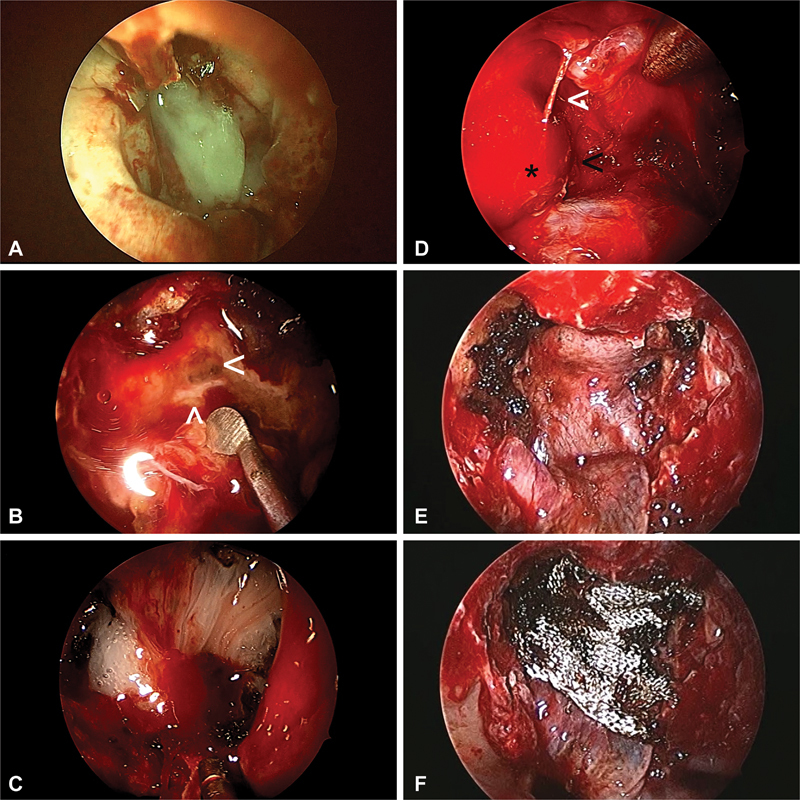
( **A–C** ) A case of sellar, suprasellar secreting pituitary macroadenoma, resected by transsellar approach; however, there was a small rent in the tuberculum dura causing cerebrospinal fluid (CSF) leak while cauterizing a superior intercavernous sinus bleed. A three-layered reconstruction was done with fibrin glue alone as sealant/adhesive. The patient had postoperative CSF leak following pack removal on day five, for which a CSF leak repair revision was done using Surgicel and fibrin glue as sealant/adhesive. ( **A** ) Primary surgery where only fibrin glue was used as sealant/adhesive over the three-layered reconstruction. ( **B** ) Revision surgery showing the site of leak at the level of tuberculum sellae anterior to the superior intercavernous sinus. **Notes:** white (<), site of leak; white (^), superior intercavernous sinus. ( **C** ) Surgicel and fibrin glue were used as sealant/adhesive. ( **D–F** ) A case of sellar suprasellar growth hormone secreting pituitary macroadenoma with right Knosp 2 cavernous sinus involvement, resected by transsellar approach, three-layered reconstruction with the last layer as a Hadad flap, and then Surgicel alone used as sealant/adhesive. ( **D** ) **Notes:** black (*), right cavernous internal carotid artery; black (<), posterior compartment of cavernous sinus; white (<),superior compartment of right cavernous sinus.

**Fig. 3:**
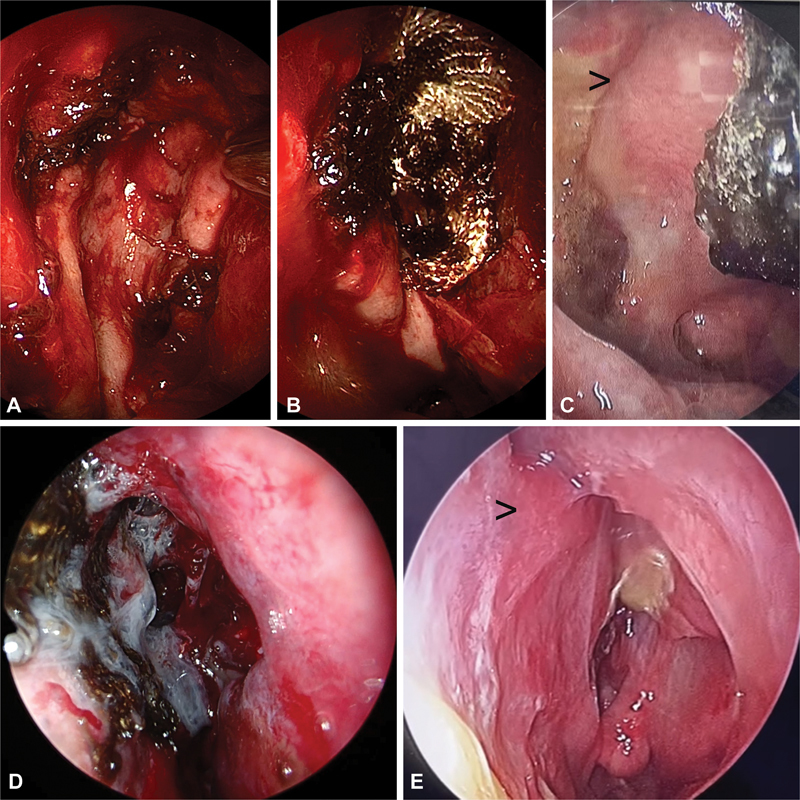
( **A,B** ) Hadad flap in position as a third layer in a transsellar approach and secured with Surgicel alone as sealant/adhesive. ( **C** ) Three weeks postoperative picture. **Note:** black (>), showing the incorporation of the margins of the flap. The mold of crust can be seen separating on its own. ( **D** ) A 1.5-cm left cribriform defect with spontaneous cerebrospinal fluid leak reconstructed with three layers and held in position with Surgicel and fibrin glue. ( **E** ) Six weeks postoperative picture. **Note:** black (>), shows flap incorporation and mucosalization.

**Fig. 4:**
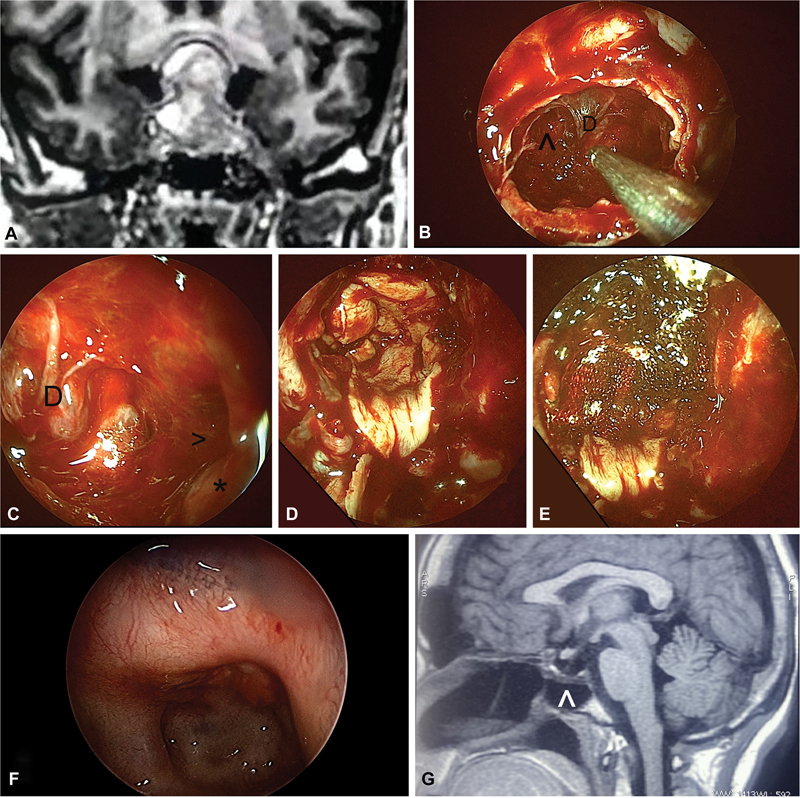
( **A** ) – Magnetic resonance imaging (MRI) coronal view of a case of growth hormone secreting sellar, suprasellar pituitary macroadenoma with Knosp 2 left cavernous sinus involvement. ( **B** ) Post complete excision of tumor. **Abbreviation:** D, descended diaphragm. **Note:** Black (^), small rent in the diaphragm due to removal of tumor firmly adherent to the diaphragm. ( **C** ) Left cavernous sinus. **Notes:** black (*), left cavernous ICA; black (>), superior compartment of left cavernous sinus. ( **D,E** ) Three-layered reconstruction with Surgicel alone as sealant/adhesive. ( **F** ) Six months postoperative endoscopic view showing complete mucosalization. ( **G** ) Six months postoperative MRI showing. **Note:** white (^), flap incorporation, descended normal pituitary gland, and stalk.

**Fig. 5:**
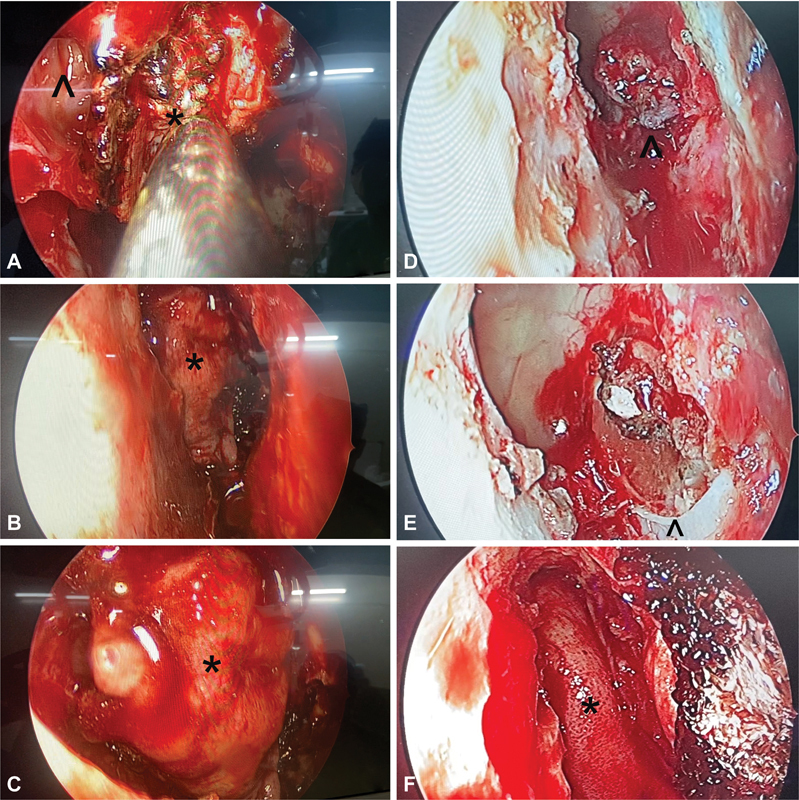
( **A** ) - A case of bilateral spontaneous cerebrospinal fluid (CSF) leak with complete cribriform plate defect. **Notes:** black (*), cribriform defect; black (^), right frontal sinus. ( **B,C** ) Three-layered reconstruction with fat, fascia lata, and Hadad flap with Surgicel alone used as sealant/adhesive. ( **D** ) Posttraumatic right side posterior table of frontal sinus encephalocele with active CSF leak. Note: black (^), encephalocele. ( **E** ) Encephalocele reduced. **Note:** black (^), margins of bony defect. ( **F** ) Three-layered reconstruction with Surgicel alone as sealant/adhesive.

As a routine procedure, the Hadad flap was harvested at the beginning of all endoscopic endonasal skull-base procedures in which a dural defect was anticipated. Once the defect was delineated in cases of CSF leak or after tumor excision in cases of skull-base tumors, the surrounding bone of at least 1 cm was denuded of the mucosa to expose the raw bone for adequate graft and flap adherence. In most cases, triple-layered reconstruction with fat, fascia lata, and Hadad flap was conducted. The fat was usually used as an underlay free graft, fascia lata as an overlay-free graft extending beyond the margins of the bony defect, and finally, the Hadad flap was placed over the fascia graft ( Fig. 1F ). In three cases an additional layer of septal cartilage graft was placed between the fat and fascia since it was a high-flow leak with communication with the third ventricle. In two cases a single layer of Hadad flap was placed since the defect was small. In cases where fibrin sealant was used as a single adjunct, it was applied after the placement of the Hadad flap as a final layer ( Fig. 2A ). In cases where both fibrin sealant and ROC were used, a single layer of ROC was applied over the fat, fascia and finally, the Hadad flap. The fibrin sealant was then applied over the final layer of ROC ( Figs. 2C , 3D ). In cases where only ROC was used, a single layer of ROC was applied over the fat, fascia lata, and finally, the Hadad flap ( Figs. 1F–G , 2E–F , 3A–B , 4 and 5 ). No other materials were used during the reconstruction of the skull-base defects. In all the cases, a Merocel (Medtronic plc, Minneapolis, MN, USA) pack was applied to hold the reconstruction in position and to prevent postoperative bleeding, which was routinely removed on the fourth postoperative day. All the patients were given oral acetazolamide 250 mg, twice a day for one month postoperatively.

The patients with postoperative CSF leak were initially managed conservatively with oral acetazolamide and lumbar drain for 1 week. Those patients who did not respond to conservative measures underwent revision leak repair. One patient required a permanent CSF diversion procedure (thecoperitoneal shunt) with leak repair revision.

### Statistical Analysis

All statistical analyses were performed using the SPSS Statistics for Windows software (SPSS Inc., Chicago, IL, USA), version 17.0. Descriptive statistics were presented as numbers and percentages. The Pearson Chi- squared test and odds ratio with 95% confidence interval (CI) were used for qualitative data. A two-sided *p* -value < 0.05 was considered statistically significant.

## Results

We included 64 patients in the study. The most common age groups were 21 to 40 years (46.9%) and 41 to 60 years (46.9%). In total, 35 (54.7%) patients were female and 29 (45.3%) were male ( Table 1 ).

**Table 1:** Demographic and clinical characteristics of the study patients

Characteristics	Frequency ( *n* = 64)	Valid percentage (%)	Cumulative percentage (%)
**Age (years)**			
< 20	2	3.1	3.1
21–40	30	46.9	50
41–60	30	46.9	96.9
61–80	2	3.1	100
**Gender**			
Male	29	45.3	45.3
Female	35	54.7	100
**Type of intraoperative CSF leak**			
High-flow leak	21	32.8	32.8
Low-flow leak	43	67.2	100
**Size of bony defect**			
Less than 1 cm	22	34.4	34.4
1–2 cm	28	43.7	78.1
More than 2 cm	14	21.9	100
**Approach**			
Transfrontal	2	3.1	3.1
Transcribriform	23	35.9	39.1
Transsellar	14	21.9	60.9
Transplanar tubercular sellar/transplanar tubercular	21	32.8	93.8
Transclival	3	4.7	98.4
C1–C2 approaches (odontoidectomy)	1	1.6	100
**Tissue layer**			
Fat	64	100	100
Fascia lata	64	100	100
Cartilage	3	4.7	4.7
Hadad flap	62	96.9	96.9
**Sealant/adhesive type**			
Fibrin glue	14	21.9	21.9
Both fibrin glue and Surgicel	26	40.6	62.5
Surgicel	24	37.5	100
**Postoperative CSF leak**			
Immediate	9	14.1	14.1
Delayed	1	1.6	15.7
No leak	54	84.3	100

**Abbreviation:** CSF, cerebrospinal fluid.

### Type of Approach and Intraoperative CSF Leak

The transcribriform approach was performed in 23 (35.9%) patients, transplanar tubercular or transplanar tubercular sellar in 21 (32.8%), and transsellar in 14 (21.9%) patients. The transclival approach was performed in 3 (4.7%) patients, transfrontal in 2 (3.1%) patients, and C1–C2 approach in 1 (1.6%) patient. Low-flow intraoperative CSF leak was observed in 43 (67.2%) patients, and high-flow leak was detected in 21 (32.8%) patients ( Table 1 ).

### Type of Reconstruction and Sealant/Adhesive Used

In 59 (92.2%) patients, a 3-layered reconstruction was performed with fat as the inlay, and fascia lata as the overlay, which was followed by the Hadad flap placement. In 3 (4.7%) patients, an additional layer of nasal septal cartilage was used between the fascia lata and Hadad flap in a gasket seal fashion. Initially, fibrin glue alone was used as a sealant/adhesive in 6 (10.71%) patients. Later, we used both ROC and fibrin glue to hold the reconstruction in place in 26 (40.6%) patients. Later, ROC alone was used as a sealant/adhesive in 24 (37.5%) patients. Recently, fibrin glue alone was used again in 8 (12.5%) patients ( Table 1 ).

### Postoperative CSF Leak

Ten (15.6%) patients had postoperative CSF leak, wherein 9 (14.1%) had immediate postoperative leak (within 1 week of surgery) and 1 (1.6%) patient had delayed leak ( Table 1 ). In 4 (40%) patients, the CSF leak resolved with conservative management, whereas 6 (60%) patients required revision surgery for closure of the leak. In our initial phase of learning curve in which we used fibrin glue alone, 4 (66.67%) of the 6 patients had postoperative CSF leak. Consequently, in view of the high incidence of postoperative leakage, we changed to a combination of both ROC and fibrin glue. The postoperative CSF leak rate was reduced to 7.7% (2 out of 26 patients). Subsequently, we used ROC alone, and the postoperative CSF leak rate was 8.3% (2 out of 24 patients). Considering the possibility of the beginning of the learning curve interfering with the outcome of the initial fibrin glue group, we recently performed reconstruction in another 8 patients with fibrin glue alone as sealant, and the postoperative CSF leak rate was 25% (2 out of 8 patients). On comparative analysis of the 4 different groups by the Pearson Chi-squared test the *p* -value was 0.002, indicating a better outcome when a combination of fibrin glue and Surgicel or Surgicel alone were used ( Table 2 ). The odds ratio for the fibrin glue alone group, in the beginning of the learning curve, was 17.33 (95% CI, 2.603–115.438), with A *p* -value of 0.000. The fibrin glue and Surgicel group had an odds ratio of 0.313 (95% CI, 0.061–1.611), with a *p* -value of 0.148. Subsequently, the Surgicel alone group had an odds ratio of 0.364 (95% CI, 0.070–1.878), with p value of 0.213. Finally, when fibrin alone was used later in the learning curve, the odds ratio was 2.000 (95% CI, 0.342–11.703), with p value of 0.435 ( Table 3 ). When a combination of both fibrin glue and Surgicel or Surgicel alone was used there were comparatively better outcomes with respect to prevention of postoperative CSF leak than using fibrin glue alone, though no individual group showed a statistically significant preventive effect. Fibrin glue provided an intraoperative watertight seal; however, the effect was short lasting. ROC showed an effective intraoperative and long-term postoperative sealant and adhesive activity in holding the reconstruction intact.

**Table 2:** Statistical comparison of the sealant/adhesive types in terms of postoperative cerebrospinal fluid leak

			Postoperative CSF leak		Total	Chi-squared test *p* -value
			Present *(H/L)	Absent *(H/L)		
**Type of sealant**	**Fibrin glue alone (beginning of learning curve)**	Count	4 (0/4)	2 (0/2)	6 (0/6)	Pearson Chi-squared = 14.599 ***p*** **-value = 0.002**
		% within type of sealant	66.7%	33.3%	100.0%	
	**Both fibrin glue & Surgicel**	Count	2 (1/1)	24 (8/16)	26 (9/17)	
		% within type of sealant	7.7%	92.3%	100.0%	
	**Surgicel alone**	Count	2 (2/0)	22 (8/14)	24 (10/14)	
		% within type of sealant	8.3%	91.7%	100.0%	
	**Fibrin glue alone (later in the learning curve)**	Count	2 (2/0)	6 (⅕)	8 (⅗)	
		% within type of sealant	25.0%	75.0%	100.0%	
**Total**		Count	10 (5/5)	54 (17/37)	64 (22/42)	
		% within type of sealant	15.6%	84.4%	100.0%	

**Abbreviation:** CSF, cerebrospinal fluid.

Note: *(H/L) – H (cases with intraoperative high-flow leak) / L (cases with intraoperative low-flow leak).

**Table 3:** Statistical outcome of each of the sealant/adhesive types in terms of postoperative cerebrospinal fluid leak

		Postoperative CSF leak	
		Odds ratio (95% CI)	Pearson Chi-squared test *p* -value
**Type of sealant**	**Fibrin glue alone (beginning of learning curve)**	17.33 (2.603–115.438)	0.000
	**Both fibrin glue and Surgicel**	0.313 (0.061–1.611)	0.148
	**Surgicel alone**	0.364 (0.070–1.878)	0.213
	**Fibrin glue alone (Later in the learning curve)**	2.000 (0.342–11.703)	0.435

**Abbreviations:** CI, confidence interval; CSF, cerebrospinal fluid.

## Discussion

In this study, we aimed to investigate the role of ROC (Surgicel) as a sealant and adhesive in addition to its hemostatic activity. This material is commonly used as a hemostatic agent during reconstruction in skull base surgery; however, its role as a sealant/adhesive is often overlooked and has not been mentioned in literature.

The twenty-first century has brought about significant advancements in EEAs which have revolutionized the management of complex and extensive lesions of the skull base. 13 14 15 The reconstruction of the large defects arising from such extensive approaches as well as separation of the arachnoid space from the sinonasal tract are a major challenge and hindrance to the use and acceptance of EEAs. The reconstruction of small defects of the ventral skull base was reliably achieved using varied techniques with a success rate of more than 95% in various clinical series before the advent of vascular pedicled flaps. 16 17 18 The advent of pedicled nasoseptal flaps has revolutionized the reconstruction of large skull base defects and thus, paved the way for EEAs. 5 6 7 8

In EEA, primary suturing of the dura of the skull base may be difficult and sometimes impossible to achieve. To minimize the risk of CSF leaks in such cases, various approaches have been used, including the use of fibrin sealants to achieve watertight closure, flap adherence and prevention of perioperative or postoperative CSF leakage. 12 Commercially available fibrin sealants contain two main active ingredients, namely, fibrinogen and thrombin, which form a fibrin clot when mixed. 19 Fibrin sealants have been reported to control the postoperative CSF leak in various studies involving transsphenoidal approaches. 20 21 22 However, the fibrin sealants were used in numerous ways, including with sutured or non-sutured patches, hemostatic agents (such as Surgicel and Gelfoam [Pfizer Inc., New York, NY, United States]), and several autologous tissues. Hence, a beneficial effect directly from the usage of fibrin sealants is difficult to confirm. 19 A large randomized Control Trial and a recent prospective trial showed no significant benefits of using fibrin sealants for preventing postoperative CSF leaks; however, some benefits were reported in terms of attaining intraoperative watertight dural closure. 23 24 A retrospective analysis of patients who underwent endoscopic repair of high-flow CSF leaks using the Hadad flap (a workhorse flap) showed no significant benefit of fibrin sealants in preventing postoperative CSF leaks. The study also concluded that this approach might significantly increase surgical costs. However, the study had also used Surgicel between the layers as well as over the Hadad flap to hold the reconstruction in place. 1

All these findings indicate that although fibrin sealants provide a watertight seal intraoperatively, they do not provide any additional benefit in terms of reducing postoperative CSF leaks. Most studies that showed benefits of fibrin sealants have invariably used additional adjunct materials, among which ROC was most commonly used. Similarly, studies that concluded that fibrin sealants are not beneficial have also used ROC to hold the reconstruction in position.

Regenerated oxidized cellulose, when applied over fat, is an excellent conduit for molding the fat into the defect. It also acts as a good sealant/adhesive for adhering the fascia graft and Hadad flap to the underlying bone. It forms a thick, dark crust-like mold that holds the reconstruction in place and prevents CSF permeation until complete healing occurs, and later, separates on its own ( Figs. 1H and 3C, E )

A recent study on the safety profile of ROC in the human respiratory mucosa showed that ROC-treated mucosa exhibited a higher incidence of cell necrosis and epithelial cell detachment. However, the study was limited in that they could not assess the effect of ROC on the deep layers that may contribute to healing. Furthermore, the role of the immune system or innate pH buffering mechanisms could not be assessed because the exposure duration in the study was of only 15 minutes. Moreover, the study could not assess whether the initial inflammatory response induced by ROC could benefit the healing process. Lastly, no necrosis of the flap edge was detected, even though the flap edge is the common site of leak. 25 In our study, we observed that ROC causes initial inflammatory response and crusting, with no significant effect on healing in the long term ( Figs. 3C, E and 4F ).

An in-vivo study on wound healing in rats with rectus abdominis muscle defect showed that despite the increased inflammatory reactions caused by Surgicel, the overall wound healing process was not significantly affected. 26

Flap necrosis was not observed in any of our patients after primary repair, with only one patient exhibiting delayed flap necrosis with CSF leak 3 weeks postsurgery. However, this case involved a revision pituitary macroadenoma excision in which an adequate flap was not available. Hence, the flap was harvested from the remaining inferior aspect of the septum and floor of the nasal cavity, which might have led to detrimental effects on the healing of the patient.

Regenerated oxidized cellulose seems to be an effective and malleable conduit for holding the layers of reconstruction in position, albeit with some immediate inflammatory changes but no notable deleterious effects on wound healing. Currently, ROC does not have FDA approval for use as a sealant or adhesive. However, it is used by most skull-base teams worldwide for holding the reconstruction in place in addition to its hemostatic effect. Moreover, Surgicel is a cost-effective alternative to fibrin glue.

This study had some limitations since it was a retrospective analysis due to the paucity of these cases. The individual agents did not show statistically significant control of postoperative CSF leak. However, ROC provided an effective intraoperative and long-term adhesive and sealant effect when compared with fibrin glue, which provided effective intraoperative watertight seal but failed to provide long-term support. Fibrin glue had early resorption compared with ROC. The authors would recommend large multicenter trials during the same time frame to support these findings.

## Conclusion

Regenerated oxidized cellulose is an excellent cost-effective substance in endoscopic endonasal reconstruction of skull-base defects with both hemostatic and sealant/adhesive effects compared with fibrin glue and can be used for holding the reconstruction in position until complete healing occurs. Although some short-term inflammatory reactions are induced by ROC, they do not have notable deleterious effects on long-term healing. A large series of randomized controlled studies is required to further support these findings.
